# Learning from older peoples’ reasons for participating in demanding, intensive epidemiological studies: a qualitative study

**DOI:** 10.1186/s12874-017-0439-9

**Published:** 2017-12-12

**Authors:** Alicja M. Baczynska, Sarah C. Shaw, Harnish P. Patel, Avan A. Sayer, Helen C. Roberts

**Affiliations:** 1 Academic Geriatric Medicine, University of Southampton, Southampton, UK; 2 National Institute for Health Research Southampton Biomedical Research Centre, University of Southampton and University Hospital Southampton NHS Foundation Trust, Southampton, UK; 3 National Institute for Health Research Collaboration for Leadership in Applied Health Research and Care, Wessex, University of Southampton, Southampton, UK; 4 MRC Lifecourse Epidemiology Unit, University of Southampton, Southampton, UK; 5 AGE Research Group, Institute of Neuroscience, Newcastle University, Newcastle, UK; 6 National Institute for Health Research Newcastle Biomedical Research Centre, Newcastle upon Tyne Hospitals NHS Foundation Trust and Newcastle University, Newcastle, UK

**Keywords:** Motivation, Patient participation, Qualitative research, Aged, Epidemiological study

## Abstract

**Background:**

Recruitment rates of older people in epidemiological studies, although relatively higher than in clinical trials, have declined in recent years. This study aimed to explore motivating factors and concerns among older participants in an intensive epidemiological study (Hertfordshire Sarcopenia Study - HSS) and identify those that could aid future recruitment to epidemiological studies and clinical trials.

**Methods:**

Participants of the HSS fasted overnight and travelled several hours each way to the research facility at an English hospital for extensive diet/lifestyle questionnaires and investigations to assess muscle including blood tests and a muscle biopsy. We conducted semi-structured interviews with 13 participants (ten women) at the research facility in May–October 2015. The interviews were audio-taped, transcribed verbatim, coded and analysed thematically by three researchers.

**Results:**

We identified personal motives for participation (potential health benefit for self and family; curiosity; comparing own fitness to others; socialising). Altruistic motives (benefit for other people; belief in importance of research) were also important. Participants voiced a number of external motives related to the study uniqueness, organisation and safety record; family support; and just ‘being asked’. Anxiety about the biopsy and travel distance were the only concerns and were alleviated by smooth and efficient running of the study.

**Conclusions:**

Personal and altruistic reasons were important motivators for these older people to participate in demanding, intensive research. They valued belonging to a birth cohort with previous research experience, but personal contact with the research team before and after consent provided reassurance, aided recruitment to HSS and could be readily replicated by other researchers.

Any fears or concerns related to certain aspects of a demanding, intensive study should ideally be explored at an early visit to establish a good relationship with the research team.

## Background

Participation rates of older people have traditionally been relatively high in epidemiological studies such as birth cohorts (95% in Cambridge City over-75 s Cohort, [1] 59% in Newcastle 85+ Cohort Study, [2] 53% in Hertfordshire Cohort Study [3]), with many participants in their 70s and 80s continuing to participate in successive rounds of data collection, and attrition mainly caused by mortality and morbidity. By contrast, in many clinical trials older people are recognised to be under-represented [4]. This may reflect trial design with an upper age limit or exclusion criteria including polypharmacy, specific co-morbidities or physical disability. Patient factors may also lead to under-recruitment and may be addressed through a focus on the specific needs of older people, for example relating to sensory impairment, mobility problems, fatigue, carer involvement, transport issues and fixed daily home routines [5]. Importantly, the result of this under-representation is that clinical trial results may not be applicable to older patients, depriving them of potential treatment benefits or exposing them to unidentified adverse effects [5].

Participation rates, although higher in epidemiological studies, have also declined over the last 30 years for a number of reasons including the more demanding, intensive nature of modern research [6]. This may represent a threat to the generalisability of the results and researchers have attempted to explore the characteristics of non-participants [7]**.**

Learning about barriers and motivations to participation can help researchers to improve recruitment especially into more demanding or time consuming studies [8]. Participants in clinical trials may expect to access new medications, technologies and better care [9, 10] whereas in epidemiological studies personal or family experience of the studied illness as well as belief in public interest are reported to be important motivations [11]. There is evidence that among younger or mixed adult age populations, participation is a dynamic, socially complex process that involves various degrees of altruism and expectation of personal gain rather than a simplistic division between self and other oriented motivations [10]. However, older people’s motivation to participate in demanding or invasive studies has been little investigated. This study aimed to explore motivating factors and concerns among older participants in an intensive epidemiological study and identify those that could aid future recruitment to epidemiological studies and clinical trials.

## Methods

The Hertfordshire Sarcopenia Study (HSS) is an epidemiological cohort study designed to investigate life-course influences on muscle morphology, mass and strength in community dwelling older people [12]. It is a sub-study of the Hertfordshire Cohort Study, a well-established birth cohort consisting of 2997 community dwelling men and women born in Hertfordshire between 1931 and 1939 [3]. Consent was sought from General Practitioners to ascertain the appropriateness of their patients taking part in the study. After receiving consent to approach members of the Hertfordshire Cohort, invitation letters as well as detailed study information sheets were sent out. Those who expressed interest were telephoned and screened for exclusion criteria by a member of the research team. Potential participants were later visited at home by the study doctor who assessed their suitability, explained the study in detail and obtained written informed consent. Participants attended the research clinic on 1 day during which they agreed to undergo a muscle biopsy after an overnight fast and a 3–4 h taxi journey to and from the research facility, leaving home around 7 am and returning around 7-8 pm. Data collected within the study are presented in Table 1. Participants’ GPs were notified of the results of dual energy X-ray absorptiometry (DXA) scan, blood pressure and fasting blood sugar [13]. Out of 1521 of older people aged 76–84 years invited to participate 274 were recruited to HSS (18% recruitment rate). The reasons for non-participation included no reply to the invitation letter or a refusal, exclusion at the time of telephone screening, or exclusion at the time of home visit (on the basis of eligibility criteria such as neurological conditions affecting the muscle, illness precluding informed consent or ability to travel and anticoagulant drug use).

**Table 1:** Data collected from participants in this study

Blood pressure and heart rate
Fasting blood test
Urine test
Tests of muscle strength and endurance
Anthropometry
Dual energy X-ray absorptiometry (DXA) scan
Peripheral quantitative computerised tomography
Cognitive function tests
Extensive health and lifestyle questionnaires
Muscle biopsy (vastus lateralis)

Thirteen participants (ten women) were recruited prospectively from a total of 15 women and three men who attended the research clinic at the hospital clinical research facility on 7 days in May, June, July and October 2015. All participants who were asked agreed to take part and the remaining five women were not asked to participate due to reaching data saturation. The small number of men in this study reflects the sampling method of the HSS (initial recruitment of 105 men followed by recruitment of 169 women and men). There was no evidence of a seasonal effect on HSS recruitment, but clinic dates were arranged to avoid bank holidays and summer breaks.

The interviews were based on a semi-structured interview schedule (see Table 2) developed by the researchers after reviewing relevant literature. One researcher (AB) conducted all of the interviews in a private room in the early afternoon after the biopsies and most assessments were completed. Usually, only the assessments of walking speed or standing balance were still outstanding for one of the interviewees by that point. The interviews were not cut short or interrupted by other assessments and lasted on average 6 min. The interviews were audiotaped and transcribed verbatim and each transcript was identified by a study number only. The interviews were conducted until no more new information was emerging.

**Table 2:** Interview schedule

**Demographic/background data**
1. How old are you? 2. Do you have a spouse? 3. Do you have any children/grandchildren? 4. How active are you? Do you do any regular exercise? 5. Co-morbidities
**Initial thoughts** Could you describe how you found out about the study? What were your thoughts initially? What appealed to you most?
**Third party role** Have you spoken to anyone about the study? Did they encourage or discourage you?
**Motivators/advantages** How did you decide that you want to take part? What are the advantages of taking part in this study? (consider personal, familial, wider societal benefits)?
**Fears/concerns** What were your fears/concerns about taking part? How have you dealt with them?
**Barriers to participation** Do you have any conditions or illnesses that make participation in this study troublesome or inconvenient?
**Impression of the study** What is your impression of the study so far? Are there any aspects you liked or disliked in particular? (ask about phone calls, home visit, journey, venue, staff, procedures, assessments, scans, meals) Would you be happy to participate again if asked?
**Other studies** Have you taken part in any other studies (within Hertfordshire Cohort Study or not)?

### Ethics, consent and permissions

The study was approved by the Hertfordshire Research Ethics Committee (REC reference 07/Q0204/68). The participants gave written informed consent to participate in this qualitative study including an audio recorded interview.

### Consent to publish

The participants gave written consent to publish the results of this qualitative study.

### Data analysis

Data was analysed using Framework [14] after completion of all interviews. Framework was developed by social researchers in the late 1980s and has been since widely adopted for use in health research [15]. In summary, the method can be divided into 7 stages. Stage 1 Transcription of the audio recordings; Stage 2 Familiarisation with the interview; Stage 3 Coding which involves applying a label (‘a code’) that describes a particular passage of importance and may be ‘open’ (without any predefined concepts) or based on pre-existing theory or identified areas of interest. This should be performed independently by at least two researchers. Stage 4 is developing a working analytical framework which should result from a consensus reached by the researchers after coding the first few transcripts and is created by grouping the codes into broader categories. Stage 5 is applying the analytical framework to all subsequent transcripts (i.e. indexing). Stage 6 Charting data into the framework matrix which involves summarising the data by category from each transcript; and finally Stage 7 Interpreting the data.

In this study, open coding was not used due to the deductive nature of the study and an a priori coding framework was developed by two researchers (AB and SS) based on the interview schedule. This working analytical framework was reviewed and modified accordingly after analysing the first three interviews. It was then used for indexing of all the transcripts (including revisiting the first three) by each researcher working independently. Regular meetings took place during the process and any disagreements were resolved by discussion between AB and SS. The coding framework was enriched and developed further after repeated analysis of all the data with direct input from a third researcher (HR). Themes arising from the data were discussed in joint meetings.

Within the framework the following categories were created: background data (e.g. age, marital status, number of children/grandchildren, and level of fitness), initial thoughts about the study, particular motivating and demotivating factors, any concerns about participation, and general impression of the study. Interpretation of data led to identification of general themes around personal, altruistic and external motives and participant fears with specific examples from the transcripts. The analysis was aimed at identifying commonalities and differences among the participants and at eliciting a wide range of views and perspectives surrounding the topic. Therefore, opinions expressed by one participant were equally weighted as views of the majority.

## Results

### Participants

Thirteen participants were interviewed (3 men) with a mean age of 78 years (range 75–83 years). Four participants lived alone (two divorced, one widowed, one bachelor) and nine lived with a partner, all in their own accommodation. All participants reported taking regular walks, five gardened regularly and five took part weekly in physical activities including jogging, cycling, bowling, dancing, Pilates and yoga. No one declared current health problems apart from osteoarthritis (four participants).

The interview analysis elicited three main themes: 1) motivations, 2) concerns, and 3) general impression of taking part in the study.

### Motivations

Two types of motivations were identified (see Table 3): internal which included personal and altruistic reasons for participation and external which were related to the structure and organisation of the study. Most participants expressed several motives which had contributed to their decision to take part.

**Table 3:** Motivations and barriers to participation identified by study participants

**Motivations to participation** Internal motivations: - Personal reasons Checking health status Comparing own fitness/health to others Improving future health of own family Curiosity/interest Meeting other people/day out - Altruistic reasons For future generations Altruistic attitude in life Previous experience in helping older people (job related) To support research
External motivations: Awareness of being part of a cohort and therefore ‘special’ High quality and good organisation of the study Well established safety record of the study Family recognition of the study quality Being approached/asked
**Barriers to participation** Anxiety about the muscle biopsy (pain, loss of independence) Effort involved in travelling to the clinic

### Internal motivations - personal

The majority of interviewees hoped to obtain personal health benefits from participating, sometimes referring to the study as ‘an MOT’ (this is an annual UK test to check if a vehicle meets road safety and environmental standards, and the term is often used to refer to a general health check). They assumed that they would get a health check and that any issues identified would be reported back to their GP. This motive was particularly relevant for one participant who did not visit his GP regularly and was found to have hypertension which was reported to his GP. Only one female participant did not identify a personal motive.

*‘It also gives you like an MOT of your own you know physical condition. So if anything jumps out which isn’t quite normal, then it’s brought to your attention and hopefully you’ll do something about it.’* (P10).

A number of participants expressed a wish to compare their own health and fitness to others. Although no formal feedback was given to participants regarding their performance, which had been explained during the initial discussion before consent, participants wanted to find out informally from the nurses how their result compared to others.

*‘…well just to generally know how my health compares to other people of my age and whether I’m reasonably fit for my age.’* (P07).

One person hoped it might help their own family:

*‘I’ve got grandchildren I love dearly and they’ll have children hopefully, please God, and in future it could help them as they get older. To live a longer, better life; more active. Be able to do more.’* (P06).

Some viewed the study as an opportunity to socialise with others and as ‘a day out’ even though they led very active and sociable lives and had good ties with their families. This view was enhanced by the verbal feedback received at the end of the clinic when they thanked the research team for making the day so ‘enjoyable’.

*‘And just something different and meeting other people again.’* (P09).

The topic of the study itself evoked a lot of interest and being asked to participate prompted some of the interviewees to volunteer.

*‘…because I’m quite interested in, particularly now loss of muscle power, because I’d noticed it in my golf and so I don’t hit the ball as far.’* (P13).

### Internal motivations – altruistic

A major theme arising from most responses in combination with personal benefit was the expectation that the study will help improve the health of future generations in a tangible way.

*‘Because it’s going to help other people eventually you know.*’ (P03).

For one participant who had been a blood donor for 23 years taking part in this study was simply a natural continuation of life-long passion of helping others:

*‘It’s nice to help other people, if I can, if the results are going to help other people, that’s fine. You know I’ve been helping people all my life, one way and another.’* (P12).

For another participant previous exposure to older people experiencing falls in her working life as a warden encouraged participation in a study that aims to investigate loss of muscle in ageing.

*‘Well I know it’s a study to help, obviously to help future people, and more people, well more people have falls as they get older….’* (P05).

Two female participants were particularly keen to help advance research in general even if they were unlikely to benefit from any personal gain. One had worked in a research environment before she retired and therefore showed a considerable insight into importance of engaging with the study.

*‘I thought yes. I’m very happy to help, and it’s all for the future of other people. Whether I might benefit I don’t know, but I thought, I think research is the way forward in medicine.’* (P10).

*‘Well I thought if nobody did it then you wouldn’t be able to find out these things.’* (P08).

### External motivations

During the analysis it became clear that certain aspects of the study itself had prompted a positive response from the potential volunteers. First of all, being part of an established cohort with historical birth records was reported as a motivating factor by the participants.

*‘The doctor told me today something about there were about three thousand eligible persons. Of whom the birth records were kept or discovered. That’s quite a small number, so I was interested in, in being one because it was fairly unique.’* (P11).

All the participants had been previously contacted as part of the main cohort study and thus showing interest in this study was a natural continuation of their ongoing interest and contribution.

*‘They contacted me, many years ago, and explained that certain people who were born between certain years, who were born in Hertfordshire, they kept records, and would I like to take part in a survey (…) I had to go Hertford Hospital first of all, and I’ve been about (…) four times.’* (P05).

In fact, the siblings of some of the volunteers were envious of them being invited and wondered why they were not.

*‘But people are envious when I tell them. I mean I’ve got a sister, two sisters now, so that’s three, and but they weren’t born in those years you see. And they say how come you managed to get on this and that (…) I said no, well you weren’t born in the right time you see!’* (P05).

One participant was anticipating further contact following previous participation and was pleased to receive the invitation letter.

*‘Well I had been wondering if you would ever, if I would ever be approached again, and I thought, I just felt it was a good idea to do it.’* (P09).

The way the invitation letter was worded and the study explained was also an important factor.

*‘There’s something about the transparency of the way that the study is being conducted and how it impinges on me when they write a document to me and I read it.’* (P11).

In addition, information about the well-established safety of the muscle biopsy in the initial 105 men was reassuring and encouraging for the volunteers.

*‘Well I was a little bit wary, but I thought oh, I’ve got to go for it. Because other people have had it done and it was okay wasn’t it.’* (P01).

The majority of the interviewees discussed the invitation letter with other people, usually close family, who expressed enthusiasm for the study and encouraged them to take part.

*‘My son and my husband, they said go ahead mother, you get a full body check-up and it’s worth it, and my son said it’s for the future, my husband said yes, it’s good for you, health checks.’* (P06).

### Concerns about participating in the study

In the interviews the participants were asked whether they had any concerns regarding the study (Table 3). Most did not report any apprehension, even when specifically asked about the muscle biopsy. This seemed to be related to the fact that many participants had previously been in hospital to undergo investigations or operations and therefore were not worried about having a relatively small procedure.

*‘No, because I’ve been to hospital a few times’.* (P02).

One participant in particular was worried about loss of independence following the procedure in view of her ongoing caring responsibilities. However, after explanations were provided during the home visit she agreed to participate.

*‘Because before then I thought I was not going to do the muscle one, because I was a bit concerned if anything went wrong (…) I need to be active because my son has got (…) mental health problems.’* (P06).

The lack of anxiety participants reported was helped by the attitude of the research team who explained every step and kept the participants at ease.

*‘And I wasn’t a bit afraid or worried about anything, because everything was explained to me as we went along; the nurses, to the doctors, to yourself, everything; no worries whatsoever.’*(P08).

The only other concern mentioned was the travel distance from Hertfordshire to Southampton which could take up to 3–4 h each way depending on traffic.

*‘My only concern was whether I was going to feel tired and you know by the end of the day. And how I would feel. The journey is obviously going to be tedious going back.’* (P09).

Participants denied any health problems that could be a potential barrier to participation. Most had a degree of hip or knee osteoarthritis, including previous operations; however they did not regard this as an obstacle to volunteering in the study. One participant still worked and stated that she would have to take time off if the clinic happened to clash with her work schedule.

*‘I told them I was going to actually do it because I thought even if they say you can’t have a day off I would take my holiday off, because I do think it’s important.’* (P06).

### Impression of the study

When asked about their impression of the study the participants were unanimous in praising the efficiency and friendliness of the research team, the pleasant taxi drivers and the quality of food that was provided during the day. No-one identified anything that required improvement.

*‘I think it’s fantastic. And it’s so good… so much care and consideration by yourself and all the staff been given. I’ve been so well looked after. It was incredible. ‘*(P06).

When questioned if they would take part again only two participants were unsure because of the travelling and distance involved, while the majority agreed that they would.

## Discussion

We were interested to establish why older people agreed to participate in a demanding and intensive epidemiological study and identify factors that might aid recruitment to future epidemiological studies and clinical trials. In this qualitative study the participants expressed a mixture of personal motives such as potential health benefit for self and family, curiosity, socialising and altruistic motives such as potential benefit for other people and belief in importance of research. Most participants expressed at least two different motives for participation in the HSS and all but one reported a personal reason such as a health check. The social aspect of the study was appreciated by two women but not highlighted by the men. One woman emphasised the possible benefit for her own family and the study’s importance for the health of future generations was highlighted by most participants. The importance of research in general was mentioned by two women. One man and eight women discussed the study with their close families or friends who encouraged them to participate. Importantly, interviewees were motivated to take part through their previous research experience as well as belonging to a birth cohort. The only concerns identified in this study were anxiety about the muscle biopsy and the travel distance to the research facility. The participants were extremely positive about the experience and keen to be involved in research in future.

Notably, certain aspects of the study conduct appeared to be important to encourage participation that were also identified in a study by McMurdo et al. [5]. The study cohort was well maintained with regular newsletters, a dedicated website and regular locally based meetings organised by the research unit. Similarly, in a study of cancer patients, regular newsletters were also an important way of spreading information about available clinical trials [16]. Older people are recognised to value personal contact over written contact and appreciate good relationships with research teams [17]. Telephone contact by research nurse has been reported to increase recruitment rates of older people into a study of physical activity [18]. In this study, a continuing positive relationship starting with the invitation letter, through telephone screening, home visit, to the clinic day preceded by a reminder call from our nurses was vital to allay fears and ensured the best possible experience for our participants. Reminder calls have also been recommended by other researchers to confirm arrangements with older participants [5].

When recruiting older participants into studies that require a high degree of commitment and effort it is essential to establish whether the balance of associated risks and benefits corresponds with their expectations and degree of vulnerability [19]. This dilemma was carefully considered by the study doctor during the home visit especially when it became clear that some potential participants had expectations of diagnostic benefits from the biopsy procedure which would not be met. This highlights the importance of sufficient time at home or in clinic to explore the suitability of each participant and their expectations prior to consent. In broader research exploring the meaning of consent [20], the perceived risks, benefits and the risk-benefit ratio predicted whether the respondent was willing to take part in a specific survey. Being clear about societal and personal benefits as well as the absence of risks was emphasised as the means to improving survey participation.

Other aspects of the study valued by our participants such as the quality of the organisation, travel by taxi and a calm research facility with a good choice of meals certainly contributed to recruitment and positive participant experience. We offered to accommodate the need for spouse companionship during the clinic day but this was rarely required in practice. The study did not offer any financial incentive to the participants; likewise, one survey of 68 older research volunteers [21] found that only 6% were motivated by payment.

Studies of dementia [22] report similar motives including individualistic which corresponded to our personal reasons (e.g. valuable learning experience) and collectivistic, corresponding to our altruistic reasons (e.g. ‘helping through being part of something bigger’). ‘Being asked’ was identified as the best motivation for participation. All of our participants had previous experience of taking part in studies and were anticipating further research involvement. Law et al. noted that dementia research participants were not deterred by any of the potential costs or lack of obvious personal benefit, which is in accord with our results. Similarly, a qualitative study of older adults with mobility limitation reported personal education, comparison of self with others, maintaining vitality and altruism as the four main benefits of adherence to protocols [23].

The opinions of family members were often mentioned by our participants as a source of encouragement. Gaining family support has been reported to be important in recruitment of those aged 85 years and older [24]. However, only one participant emphasised a specific desire to help her family in the future. This finding clearly differs from genetic studies where familial motives stand on an equal footing with personal and societal ones [10]. In surveys investigating larger adult populations [25] and older volunteers [16, 26] scientific interest or receiving results of the study was quoted as an important motivator. This curiosity was characteristic of participants in this study which was fostered by special research updates via newsletters and meetings.

It is worth noting that although HSS was an intensive and demanding study it only required 1 day of involvement from the participants. In many other studies the expected commitment may be greater due to the multiple follow-ups over a prolonged period of time. Marcantonio et al. [8] interviewed 50 vulnerable older medical and surgical patients about participation in delirium research that would include long neuropsychological testing, blood tests, MRI and possibly a lumbar puncture. The main motivator expressed was to benefit mankind but also the opportunity to learn more about the disease, personal benefit, having something to do and socialisation, which is similar to the findings of this study.

It is recognised that recruitment to cohort studies which involve older people is relatively high (above 50%) in comparison to the low rates in clinical trials for common conditions in older age [27]. Clegg suggests that cohort multiple randomised controlled trial design may solve some of the problems encountered in current recruitment and provide researchers with a pool of older people who are keen to take part in studies. There is evidence that epidemiological research appeals more to people if initiated by trusted public institutions [11]. The fact that HSS relied on volunteers from an already well-established cohort may have contributed to the successful recruitment rate. It is likely that lessons learned from epidemiological studies could help recruitment to less popular clinical trials.

In health promoting studies of hard to reach older people the main obstacles to participation reported were tiredness, feeling too old, lack of motivation, deteriorating health, costs and lack of transportation [24]. Inconvenience as a barrier to participation (frequency of visits, travel distance, transport, parking, accessibility) has also been reported in older adults with mobility limitations [23]. Whereas, in an intensive study [8], the strongest disincentives reported were procedures (lumbar puncture and blood tests) and long interviews. Travel and the biopsy procedure were the only concerns reported by a minority of participants in this study.

This study has a number of strengths. It is one of a few published qualitative studies that have explored the motivation of older people to participate in demanding and intensive research. The study was conducted in a rigorous way with direct involvement of all of the research team. The transcripts were meticulously studied and coded to enable in-depth thematic analysis. However, the study also had some limitations. We are aware of the inherent, unavoidable bias of interviewing only those who have volunteered to take part in studies over several years and we were not able to explore the views of non-participants who declined the invitation or did not respond. While our participants were not frail or multi-morbid (a number of them had one co-morbidity of osteoarthritis but all were physically active), we believe that our conclusions are relevant to a wider sample of older people. The fact that a doctor visited participants at home may have added legitimacy to the consent process. One researcher (AB) interviewed all participants which avoided inter-researcher differences but may have introduced bias related to style and previous experience. AB was one of the two research doctors performing the muscle biopsy which could have led to participants feeling uneasy expressing criticism. However, the results fit with the uniformly positive informal feedback received from our participants throughout the study including thank you notes sent afterwards. AB paid particular attention to any nuances in the interviewee responses and body language that may have implied dissatisfaction. If there had been any implied discontent AB would have offered the participant the option of speaking to someone not directly involved in the study but no such case was identified. In some cases, there were outstanding assessments to be completed after the interviews which would therefore have not been taken into account in the responses. However, as these assessments were of similar physical intensity, performed by the same research nurses in the same environment; it seems unlikely that they would change participants’ general opinion about the study.

This study has identified several factors that could support recruitment in other research studies. Personal continuing contact through a carefully worded invitation, telephone calls and recruiting home visit could be replicated. The availability of a dedicated research facility is not universal but could be replaced with 7bespoke time, staff and clinical space specifically devoted to the participants. Provision of free transport and suitable choice of refreshments is easily reproduced and can enhance recruitment. It is also important to address the reasons which motivate older people and their expectations prior to consent by emphasising certain aspects of the study and exploring potential concerns.

## Conclusion

Personal and altruistic reasons for participation in research were important motivations for the older people in this study. External drivers relating to organisation of the study offered additional incentives. Any fears or concerns related to certain aspects of an intensive or demanding study should ideally be explored at a visit where a relationship of trust is built with the research team member. Smooth and efficient running of the study provides reassurance and alleviates anxiety contributing to a general positive experience for the participants.

## Abbreviations

DXA: dual energy X-ray absorptiometry
HCS: Hertfordshire Cohort Study
HSS: Hertfordshire Sarcopenia Study
MOT: Ministry of Transport test
